# Theodor Schwann (1810–1882)

**DOI:** 10.1007/s00415-021-10630-6

**Published:** 2021-06-02

**Authors:** Michał K. Owecki

**Affiliations:** Department of History and Philosophy of Medical Sciences, Poznań University of Medical Sciences, ul. Przybyszewskiego 37A, Poznań, Poland

**Keywords:** Theodor Schwann, Schwann cell, History of neurology

**Fig. 1:**
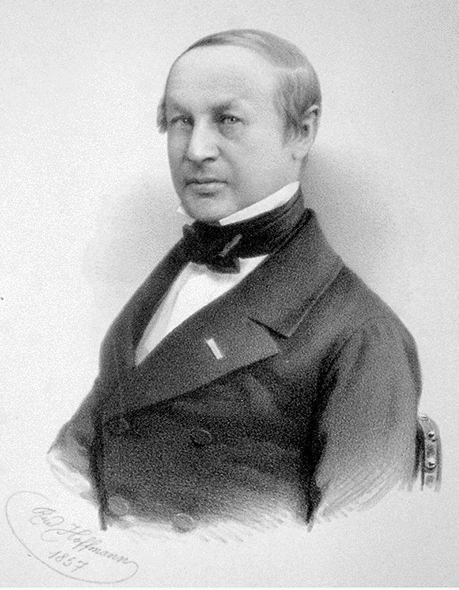
Theodor Schwann (public domain)

Theodor Schwann (Fig. 1), the eminent founder of modern histology and the discoverer of the lemmocyte, was born on December 7, 1810 in Neuss, Germany, the fourth son of Elisabeth (née Rottels) and Leonard Schwann, the owner of a local bookstore. Theodor grew up in a large family—he had twelve siblings. As a child, he proved to be multi-talented and hardworking. He initially intended to study theology, but, over time, he changed his mind and chose medicine. After graduating from the Jesuit Gymnasium in Cologne in 1829, he enrolled at the University of Bonn, where, two years later, he obtained a bachelor's degree in philosophy. It was there that he first met Johannes Müller (1801–1858), an outstanding German physiologist. Schwann not only attended Müller’s lectures, but also helped the professor in laboratory research on spinal roots. Then, in the years 1831–1833, he continued his education at the University of Würzburg, where he listened to the lectures of another illustrious scientist, Johann Schönlein (1793–1864). For the final semester of his studies he moved to Berlin, following his first mentor, Müller [1–3]. In 1834, he passed the state medical examination and obtained the title of doctor of medicine. Schwann’s dissertation *De necessitate aëris atmosphaerici ad evolutionem pulli in ovo incubato*, written in Latin, discussed chicken embryo development and was inspired and supervised by Müller, who entrusted him with the position of assistant at the anatomy museum [2, 3].

From the beginning of his career, Schwann was interested in the histology and physiology of the nervous system and muscle tissue. In his studies, he proved that the upper part of the esophagus is made of striated tissue, whereas the rest of the gastrointestinal tract, the uterus, pupils and bladder are constituted of smooth muscle. In the 1830s, he conducted a number of experiments in which he tried to make an objective determination of the force of muscle contraction under stimuli at varying intensities. His new methods made him a pioneer of neurophysiology and quantitative physiology. Unfortunately, he did not publish his results on his own—they were only cited in other medical journals from that period, including Müller’s *Handbuch der Physiologie des Menschen* and Oken’s *Enzyklopädische Zeitschrift* [3, 4].

Schwann made most of his important scientific discoveries during the Berlin period (1834–1838). In 1836 he isolated the enzyme responsible for digestive processes in the stomach—and coined the name “pepsin” for this newly identified substance. A year later, he proved that yeast fermentation activated with sugar is an expression of the life processes, and that yeasts themselves are living organisms [3, 5, 6].

In 1838, Schwann initiated a collaboration with Matthias Schleiden. The meeting of the two scientists was to have major and far-reaching consequences: the founding of cell theory [7]. According to their new idea, a single cell was the basic structural unit of every living organism. In the same year, Schwann presented his observations in a series of short articles [8]. Then, in 1839, he published an extensive work on histology: *Mikroskopische Untersuchungen über die Übereinstimmung in der Struktur und dem Wachstum der Thiere und Pflanzen* (“Microscopical Researches into the Accordance in the Structure and Growth of Animals and Plants”). A substantial part of the monograph is dedicated to the microstructure of muscles and nerves. In the book, Schwann precisely described the myelinated nerve fiber. His research led to the discovery of the cell that produces the myelin sheath that envelops the axon. In honor of his contribution, this was later eponymously named the Schwann cell [3, 7, 9]. He also found that during embryonic development individual cells unite to form the muscle fiber [10].

Schwann’s book gained international recognition after it was printed in French (in 1842) and English (in 1847). The monograph is also worth mentioning for another reason. It was in this book that Schwann coined the term “metabolism”, signifying the totality of chemical processes in a cell. The word was introduced into scientific vocabulary for the first time in the German edition of “Microscopic Researches”, and was soon adopted worldwide [7, 9].

Schwann’s undeniable successes led to a dynamic development in his career. In 1839, at the age of only 29, he accepted an offer of a professorship of anatomy at the University of Louvain, Belgium. He worked there for the following nine years. In recognition of his outstanding achievements, Schwann received the Copley Medal in 1845, the oldest and most prestigious award of the Royal Society of London [1]. In 1848, Schwann moved to the University of Liège, where he took over the professorship of anatomy and, after a few more years, that of physiology and embryology. He was then already world famous—in 1863 he was elected as an international member of the American Philosophical Society. Despite numerous offers of work from German universities (Munich, Giessen, Wrocław and Würzburg), he decided to stay in Belgium. He remained in Liège until the end of his career and it was there that he also spent the last years of his life, devoting himself to his hobby: photography. He retired in 1879, and, in the same year, was elected to the French Academy of Sciences and the Royal Society of London—a prestigious culmination of his distinguished career [1, 2].

Theodor Schwann was a gentle, calm and timid man, and one who avoided conflict. He remained a bachelor until the end of his life. Even until the formal celebrations of the 40th anniversary of his university work in 1878 in Liège, he enjoyed excellent health. Shortly before his death, however, he suffered from recurrent dizziness and anxiety attacks, diagnosed as a manifestation of heart valve disease. In December 1881, he went to Cologne to visit his family for Christmas. During this stay, he suffered a stroke, as a result of which he died two weeks later, at his brother's house, on January 11, 1882. He was buried in the local cemetery, with delegates from the universities of Bonn and Liège to bid him farewell [1–3].
